# Proton irradiation induces persistent and tissue-specific DNA methylation changes in the left ventricle and hippocampus

**DOI:** 10.1186/s12864-016-2581-x

**Published:** 2016-03-31

**Authors:** Soren Impey, Carl Pelz, Amanuel Tafessu, Tessa Marzulla, Mitchell S. Turker, Jacob Raber

**Affiliations:** Oregon Stem Cell Center and Department of Pediatrics, Oregon Health and Science University, Portland, OR 97239 USA; Department of Cell, Developmental and Cancer Biology, Oregon Health and Science University, Portland, OR 97239 USA; Department of Behavioral Neuroscience, L470, Oregon Health and Science University, 3181SW Sam Jackson Park Road, Portland, OR 97239 USA; Oregon Institute of Occupational Health Sciences and Department of Molecular and Medical Genetics, Oregon Health and Science University, Portland, OR 97239 USA; Departments of Neurology and Radiation Medicine, Division of Neuroscience ONPRC, Oregon Health and Science University, Portland, OR 97239 USA; Knight Cardiovascular Institute, Oregon Health and Science University, Portland, OR 97239 USA; Department of Pediatric, L321, Oregon Health and Science University, 3181SW Sam Jackson Park Road, Portland, OR 97239 USA

**Keywords:** Proton irradiation, Hippocampus, Ventricle, DNA methylation, RNAseq, Left ventricle, Epigenetic

## Abstract

**Background:**

Proton irradiation poses a potential hazard to astronauts during and following a mission, with post-mitotic cells at most risk because they cannot dilute resultant epigenetic changes via cell division. Persistent epigenetic changes that result from environmental exposures include gains or losses of DNA methylation of cytosine, which can impact gene expression. In the present study, we compared the long-term epigenetic effects of whole body proton irradiation in the mouse hippocampus and left ventricle. We used an unbiased genome-wide DNA methylation study, involving ChIP-seq with antibodies to 5-methylcytosine (5mC) and 5-hydroxymethylcytosine (5hmC) to identify DNA regions in which methylation levels have changed 22 weeks after a single exposure to proton irradiation. We used DIP-Seq to profile changes in genome-wide DNA methylation and hydroxymethylation following proton irradiation. In addition, we used published RNAseq data to assess whether differentially methylated regions were linked to changes in gene expression.

**Results:**

The DNA methylation data showed tissue-dependent effects of proton irradiation and revealed significant major pathway changes in response to irradiation that are related to known pathophysiologic processes. Many regions affected in the ventricle mapped to genes involved in cardiovascular function pathways, whereas many regions affected in the hippocampus mapped to genes involved in neuronal functions. In the ventricle, increases in 5hmC were associated with decreases in 5mC. We also observed spatial overlap for regions where both epigenetic marks decreased in the ventricle. In hippocampus, increases in 5hmC were most significantly correlated (spatially) with regions that had increased 5mC, suggesting that deposition of hippocampal 5mC and 5hmC may be mechanistically coupled.

**Conclusions:**

The results demonstrate long-term changes in DNA methylation patterns following a single proton irradiation, that these changes are tissue specific, and that they map to pathways consistent with tissue specific responses to proton irradiation. Further, the results suggest novel relationships between changes in 5mC and 5hmC.

**Electronic supplementary material:**

The online version of this article (doi:10.1186/s12864-016-2581-x) contains supplementary material, which is available to authorized users.

## Background

Environment epigenetics is the study of how environmental exposures interact with the epigenome to cause stable epigenetic change, most notably changes in DNA methylation patterns. Environmental exposures include physical interactions, such as chemicals and radiation [1, 2], but also extend to other interactions such as emotional stress [3] and changes in circadian rhythms [4]. Accumulating evidence has shown changes in genomic DNA methylation profiles (i.e., levels and/or distribution) from environmental exposures [1, 2], but how these exposures are translated to the observed DNA methylation changes are essentially unknown. Also unknown is whether these changes are physiological or pathological responses to the exposures, or a mixture of both.

DNA methylation is found in two main forms. The best characterized is 5 methylcytosine (5mC), which is associated with loss of transcription potential [3, 5]. No direct mechanism to remove the methyl group from cytosine has been demonstrated in mammals. A second form of DNA methylation is 5-hydroxymethylcytosine (5hmC), which is enzymatically derived from 5mC [6] by the Tet-family hydroxylases [7, 8]. The discovery of 5hmC as a modified form of 5mC changed the perception of 5mC as a stable epigenetic modification to one that may be dynamically regulated under certain conditions, including from environmental exposures [9]. The roles of 5hmC have not been fully elucidated, but current thought is that 5hmC is involved in DNA demethylation and also plays a role in active gene expression [9]. A reasonable presumption is that these two activities are linked at times.

Dynamic changes in DNA methylation are programmed during early development beginning with fertilization [10] and continue at a much lower rate after development reflecting life exposures [11–13]. Changes over the life span have reported for 5mC in human blood [14], nonhuman primate brain [15], and mouse tissues [16, 17]. The mouse studies suggested a degree of tissue-specificity [16, 17].

In the present study, we used an unbiased genome-wide DNA methylation approach to determine the extent, persistence, and tissue specificity (brain versus heart) of genomic 5mC and 5hmC changes in mice following proton irradiation, which is increasingly used in cancer therapy [18–20] and is of particular interest to NASA because protons are abundant in galactic cosmic rays and solar particle events (SPE) [21]. The brain was chosen because proton irradiation affects this tissue in humans as part of cancer treatment [22, 23], and specifically the hippocampus was chosen because it might be especially sensitive to effects of proton irradiation [24–26]. The heart was chosen as a second tissue of interest because cardiovascular (CV) disease is a latent effect from radiotherapy in cancer patients [27, 28] and has been reported in Hiroshima and Nagasaki atomic bomb survivors [29, 30].

We report persistent changes in 5mC and 5hmC in both tissues as a result of exposure, that these changes are not distributed randomly, and they reflect both tissue specific and tissue independent responses to the proton exposure. The results also suggest that the retention versus loss of 5hmC that forms from 5mC in response to proton irradiation is not random.

## Results

### DNA methylation in the left ventricle and hippocampus

**Fig. 1:**
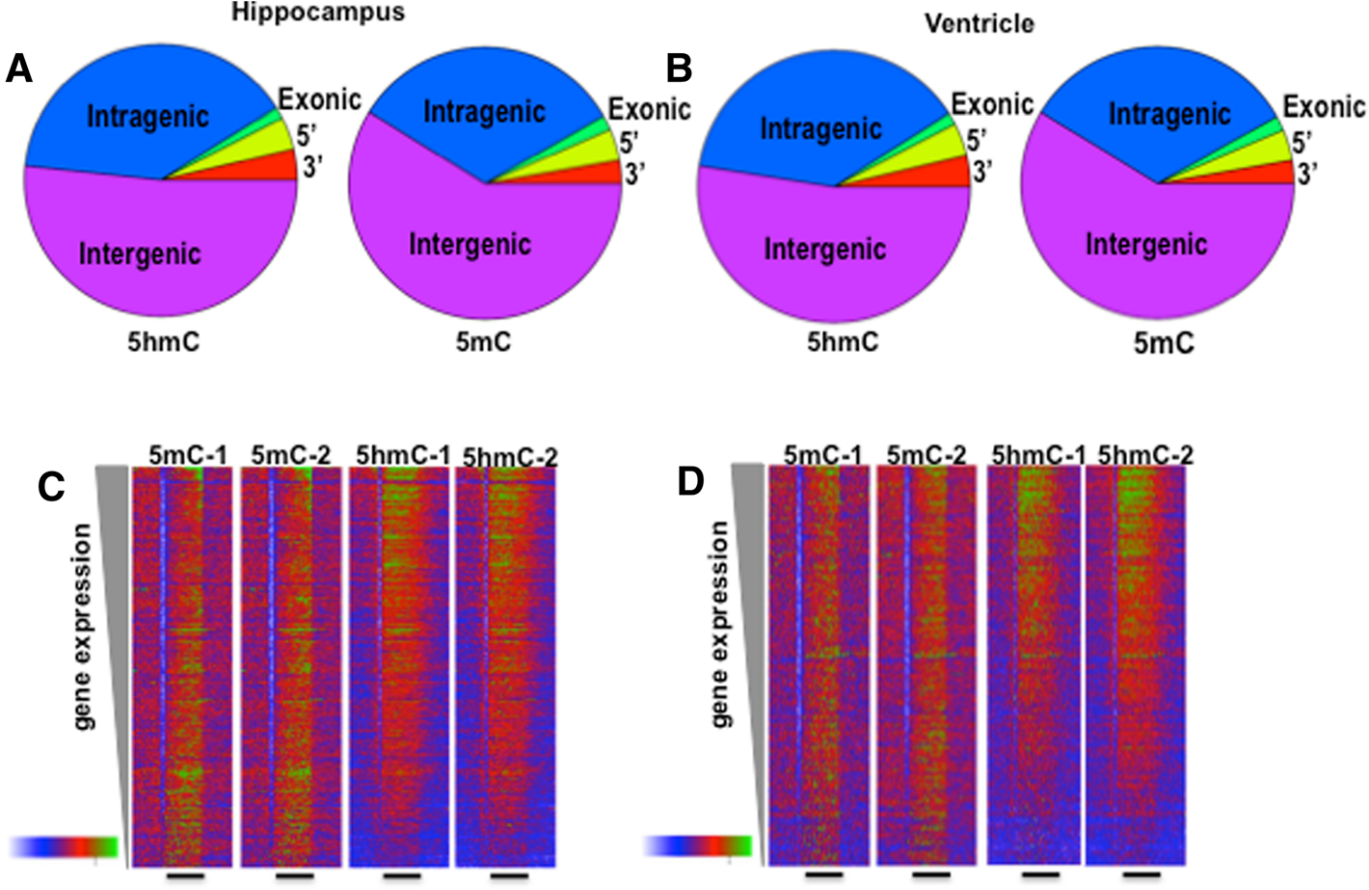
5hmC is enriched in the gene bodies of active genes while 5mC is depleted at the 5’ regions of active genes. (**a** and **b**). Pie chart depicts accumulation of 5hmC or 5mC DIP-Seq signal in the indicated genomic regions. Data that matched multiple categories was matched to the closest annotation. (**c** and **d**). Heatmaps depict DIP-Seq sequence density (500 bp bins) at RefSeq genes rank-ordered by levels of gene expression (RNA-Seq data from other studies). The color ramp shows min-max DIP/ChIP signal density normalized to the 80^th^ quantile. The black bar illustrates the transcribed region with the 5’ end on the left

To determine whether the epigenetic response to radiation injury was tissue specific, we compared DNA methylation (5mC and 5hmC) in the hippocampus and left ventricle. The tissues were removed from mice 22 weeks after irradiation with 1 Gy of protons or from sham irradiated controls. DNA from hippocampus and left ventricle was isolated and changes in the levels and distributions of 5mC and 5hmC from proton exposure were determined using me-DIP (5mC) and hme-DIP (5-hmC), respectively. We generated 8 DIP-Seq libraries (2 per tissue/radiation condition) each with greater than 30 million reads. Importantly, the 5mC and 5hmC antibodies were highly-specific with no detectable cross-reactivity by DNA dot blot (Additional file 1: Figure S1). The global distribution of 5mC and 5hmC in our unexposed hippocampal samples was remarkably similar to equivalent hippocampal samples prepared by others (Additional file 2: Figure S2 and data not shown) [31]. In addition, as expected, 5mC is enriched in poorly transcribed regions of the genome while 5hmC is enriched in highly transcribed regions of the genome in both the hippocampus and ventricle (Fig. 1a-d). Moreover, a DIP density heatmap sorted by RNA-Seq expression levels indicates the 5hmC is enriched in the gene bodies of active genes while 5mC is depleted from the transcriptional start sites (TSS) of expressed genes (Fig. 1c and d). These differences in distribution of the two epigenetic marks are consistent with other studies in brain and suggest that the two methylation marks have different biological functions [31–33]. The accumulation of 5mC or 5hmC in intragenic, intergenic, exonic, intronic, or TSS domains was not significantly different in ventricle versus hippocampus, suggesting that the global distribution of these epigenetics marks is similar across tissues (Fig. 1a-d, Fisher exact test, *p* > 0.3).

**Fig. 2:**
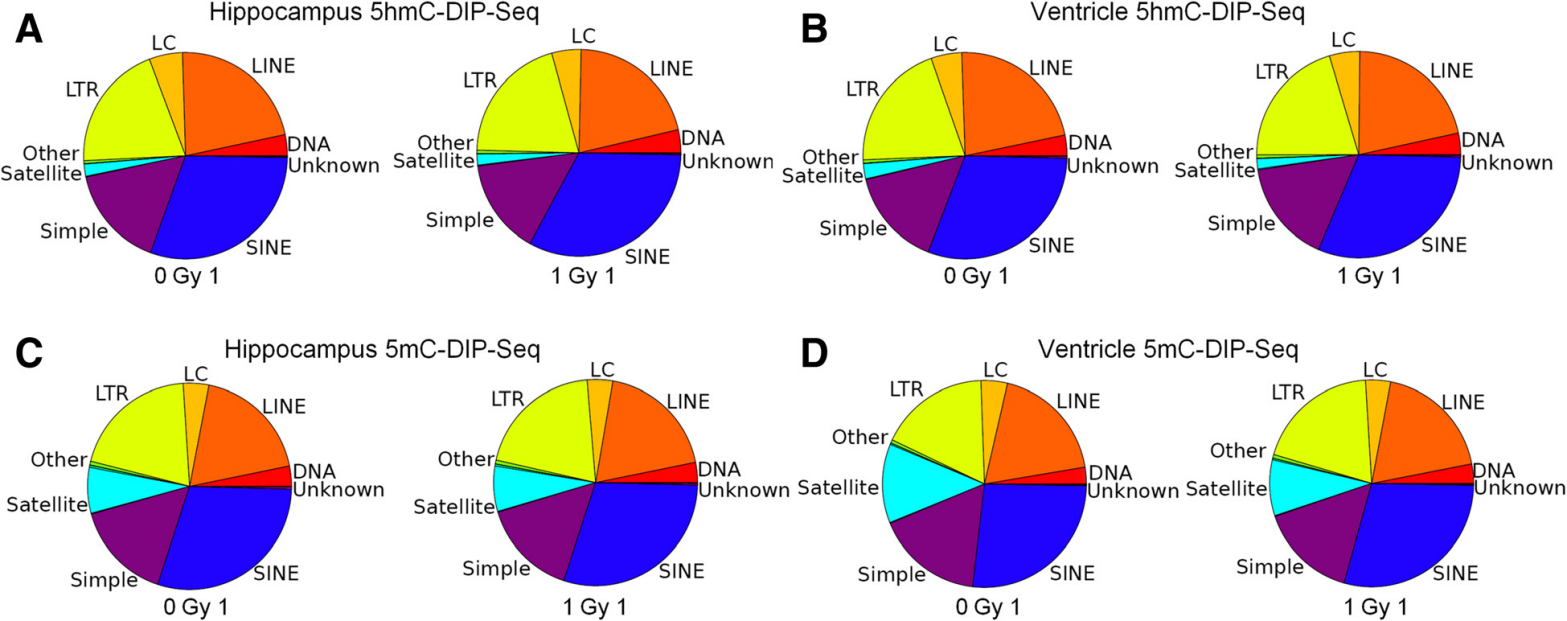
**a**-**d** Pie chart depicts accumulation of 5hmC (**a**, **b**) and 5mC (**c**, **d**) DIP-Seq signal in the indicated genomic repetitive sequence annotation (DIP-Seq counts per million repeat sequences). Data that matched multiple categories were matched exclusively to the closest annotation

Because DNA methylation has been linked to repression and organization of repetitive genomic regions we quantified the density of 5mC and 5hmC signal in RepBase repeat annotation [34]. Consistent with the well-established role for DNA methylation in maintaining stability of genomic repeats, we observed that 5mC was enriched in satellite, LINE, SINE and LTR repeat annotation [35, 36] (Fig. 2a-d). The co-enrichment for 5hmC suggests that it also plays a role in repression at these regions. There were no significant differences in 5mC and 5hmC density in repeat annotation in response to irradiation (Fig. 2a-d; *p* > 0.1).

**Fig. 3:**
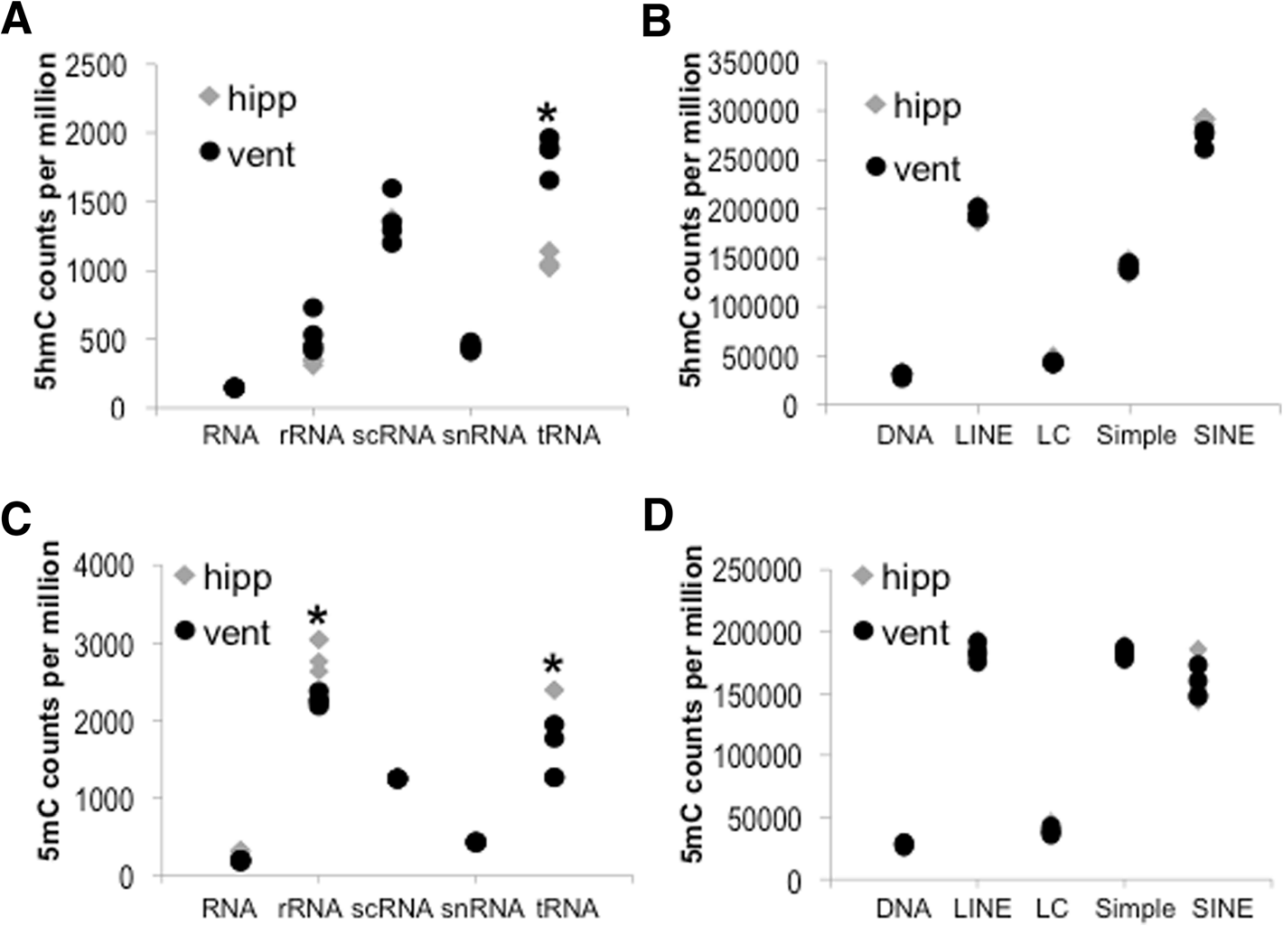
**a**-**d** Strip plots indicate the accumulation of normalized 5hmC and 5mC DiP-Seq sequences (counts per million repeat sequences). The decreased accumulation of 5mC in ventricle tRNA and rRNA repeats, as well as the increased accumulation of 5hmC in ventricle tRNA repeats, was significant (*p* < 0.05, *n* = 4)

We next asked whether differential accumulation of 5mC and/or 5hmC occurred in hippocampal and ventricular repetitive DNA (Fig. 3). Interestingly, we found 5mC counts in tRNA and rRNA genes were significantly lower in ventricular tissue than in hippocampal tissue (Fig. 3c). In contrast, 5hmC sequence counts in tRNA annotation were significantly higher in ventricular tissue than in hippocampal tissue (Fig. 3a). 5hmC sequence counts in rRNA annotation were elevated in ventricular tissue versus hippocampus but this difference was not significant (1.5 fold, *p* < 0.1). These results suggest that ventricular 5mC in tRNA and rRNA may be preferentially oxidized by Tet enzymes to 5hmC. We did not find significant tissue-specific differences in 5hmC or 5mC counts for more highly repetitive classes (Fig. 3b and d). The tissue-specific differences in 5hmC signal associated with tRNA repeats suggest that protein translation could be differentially regulated by Tet-mediated 5mC oxidation.

### DNA methylation in the left ventricle and hippocampus 22 weeks after proton irradiation

**Fig. 4:**
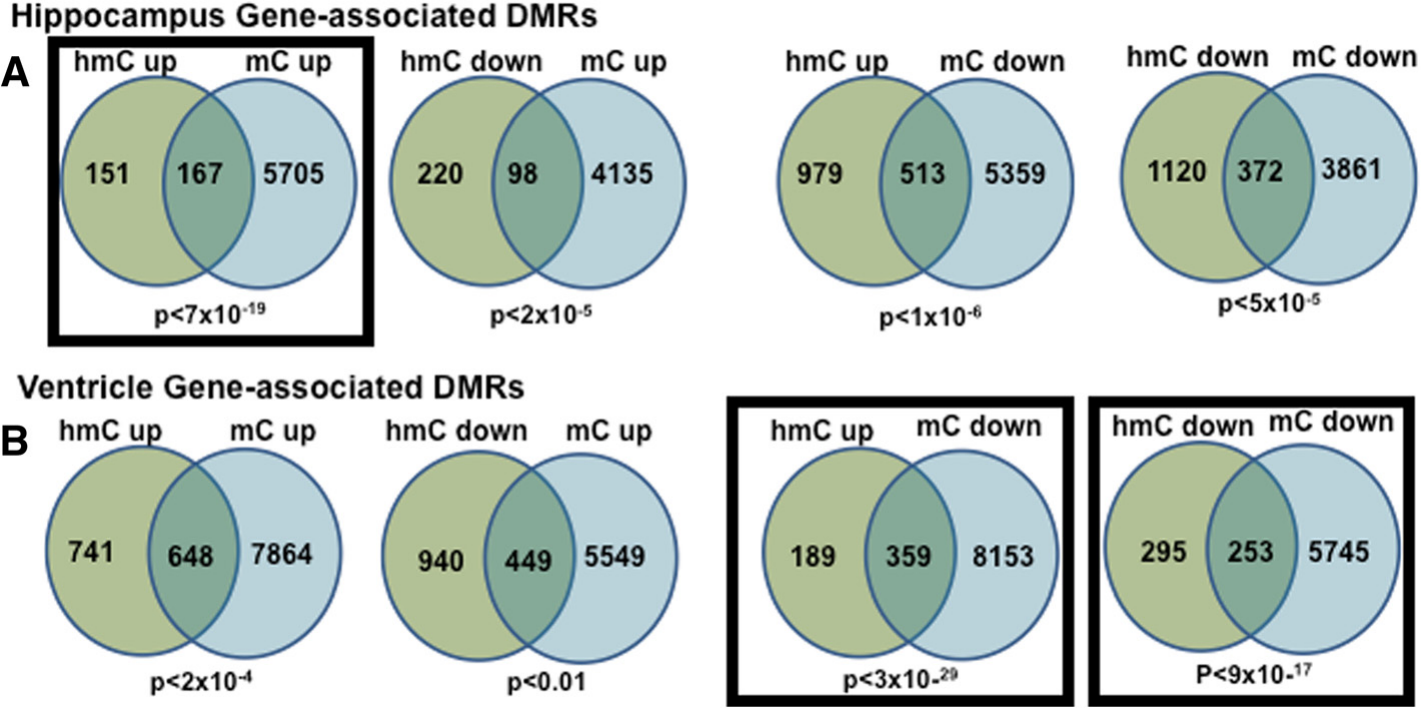
**a** and **b** Venn diagrams depict directional overlap between differentially hydroxymethylated and methylated regions (FDR-adjusted *p* < 0.01) in a 25 kb window. The three Venn diagrams with the highest level of significance for the overlap are highlighted by the black boxes they are placed in

Although global changes in gene- and repeat-associated 5mC and 5hmC signal were not significantly-regulated, unbiased analyses identified thousands of differentially-regulated methylated (DMR) and hydroxymethylated (DHMR) regions that were significantly changed by proton radiation (FDR-adjusted *p* < 0.01; Fig. 4 and Additional file 3: Table S1). Because 5hmC accumulation depends on 5mC and is believed to contribute to local demethylation (i.e., loss of 5mC), we examined the directional relationships between adjacent DMRs and DHMRs (25kb window). We use Venn diagrams to depict these relationships and highlight those with the most significant overlap (Fig. 4). In the ventricle, we found that the most significant overlap correlated increases in 5hmC with decreases in 5mC (*p* < 4x10^−29^). We also observed in the ventricle a spatial, very highly significant overlap for regions where both epigenetic marks decreased (*p* < 9x10^−17^). These results suggest that in the heart 5hmC is associated with loss of 5mC and are consistent with the conventional view that 5hmC accumulation is causally linked to local DNA demethylation.

The brain is unique among differentiated tissues because many brain regions have high levels of 5hmC that persist throughout development. In particular, up to 20 % of modified CpGs have been proposed to be 5hmC in hippocampus. Our data indicate that, in contrast to what is seen in the left ventricle, the most significant spatial correlation in the hippocampus was for regions with increased 5hmC and 5mC. Thus, these data indicate that in the hippocampus 5hmC accumulation is not causally linked to local DNA methylation. The co-localization of upregulated DMRs and DHMRs is consistent with previous observations suggesting that 5hmC is an abundant and persistent mark in brain [31, 37]. Moreover, it raises the possibility that deposition of hippocampal 5mC and 5hmC may be mechanistically coupled. And finally, the overall patterns of change for the hippocampus and ventricle suggest differences in the epigenomic responses of both tissues to proton exposure.

**Fig. 5:**
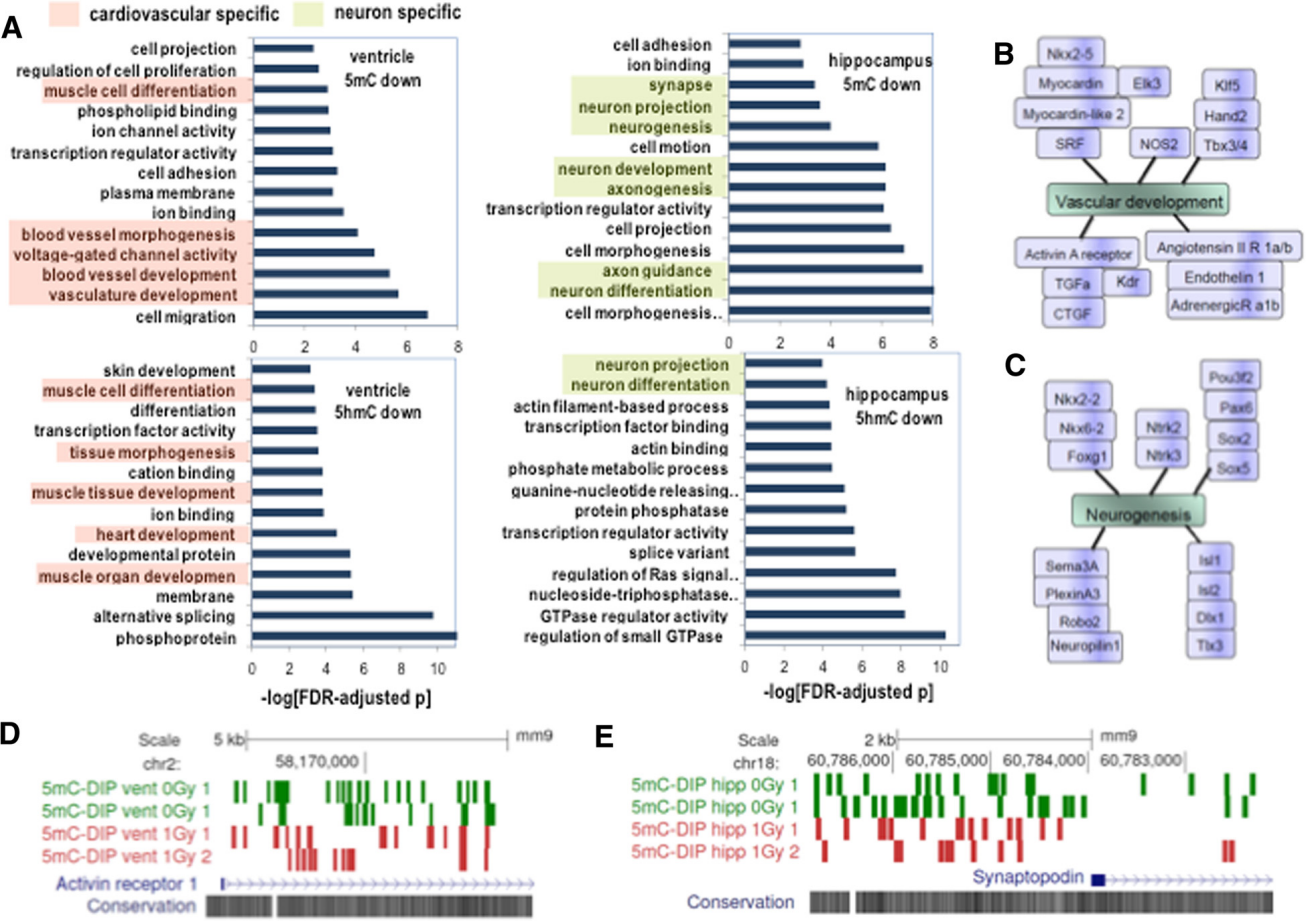
**a** Bar graph depicts gene ontology categories significantly enriched in the indicated radiation-regulated differentially methylated regions (FDR-adjusted *p* < 0.01) identified from left ventricle and hippocampus. Note the gene categories-associated with cardiovascular function (red) and neuronal function (green) are restricted to the indicated tissues. (**b** and **c**). Diagrams depict selected genes that regulate vascular development or neurogenesis that were significantly associated with decreased methylation in the indicated tissue. (**d** and **e**). UCSC genome browser diagrams depict mC-DIP-Seq signal upstream the Activin receptor 1c gene in ventricle and upstream the Synaptopodin gene in hippocampus that showed significant differences following radiation exposure. The wiggle tracks depict median-scaled tag count density above background at an FDR of 5 %. The highlighted region was statistically significant (FDR-adjusted *p* < 0.001)

To further explore the tissue specific differences and to identify biological functions for DMRs and DHRs in the response to proton irradiation, we non-redundantly annotated regions that were within 50kb of a RefSeq transcriptional start site and performed gene ontology (GO) analyses. Consistent with the finding that global methylation patterns were similar across tissues (Fig. 1a-d), GO comparisons of DMRs and DHMRs between non-irradiated tissues identified transcriptional, signaling, and metabolic pathways that likely reflect developmental processes (Additional file 4: Figure S3). In contrast, we found that proton-radiation regulated DMRs and DHMRs were markedly enriched for tissue-specific gene categories/pathways (Fig. 5a-c). In particular, the ventricle regions with decreased 5mC and 5hmC were enriched for genes linked to vascular development, ion channel activity, and muscle differentiation/development while for the hippocampus regions with decreased 5mC were linked to neuron differentiation, axon/process, outgrowth, neuron/synapse development, and neurogenesis (Fig. 5a). Diagrams depicting selected genes from enriched gene ontology pathways in heart (Fig. 5b) and hippocampus (Fig. 5c) illustrated the high-degree of redundancy. Similarly, KEGG analysis identified ventricle (calcium signaling, vascular smooth muscle contraction; Additional file 5: Figure S4) and hippocampus-associated (axon guidance; Additional file 6: Figure S5) pathway components that were significantly overrepresented in regions that had decreased 5mC. These results demonstrate tissue specific epigenomic responses to proton irradiation. UCSC genome browser diagrams illustrate the mC-DIP-Seq signal upstream of the Activin receptor 1c gene in ventricle (Fig. 5d) and upstream of the Synaptopodin gene in hippocampus (Fig. 5e) that showed significant differences following radiation exposure. The enrichment for key determinants of brain and heart cell-fate suggests that proton radiation triggered epigenetic responses that engaged or targeted tissue-specific differentiation and repair (*i.e.*, tissue response to injury). The overlapping pathway regulation of 5mC and 5hmC in heart is also suggestive of step-wise demethylation (*i.e.*, Tet-mediated oxidation following by DNA repair) linked to a radiation-induced differentiation response. Interestingly, this finding is reminiscent of the global spatial overlap between regions with decreased 5mC and decreased 5hmC in heart (Fig. 4). There was less overlap between 5mC gene pathways and 5hmC pathways (Fig. 5a and data not shown) in hippocampus, suggesting that post-mitotic neurons may have distinct epigenetic regulatory pathways.

In contrast to regions associated with decreased 5mC and 5hmC, radiation-regulated regions linked to increased 5mC and 5hmC in heart and hippocampus did not show highly significant enrichment for gene ontology categories (no categories below FDR-adjusted *p* < 0.02). Moreover, modestly significant gene categories were largely comprised of metabolic and general signaling pathways (e.g. regulation of transcription, regulation of RNA-metabolism, regulation of biosynethic process, G-protein signaling, cytoskeleton etc.). These results suggest that proton radiation-induced epigenetic remodeling induces a general transcriptional response.

## Discussion

In this study, we examined the genome-wide distribution of 5mC and 5hmC in two different tissues (left ventricle and hippocampus) from sham and proton irradiated mice using an unbiased sliding window approach to identify and merge regions enriched for these marks. Consistent with previous studies by others [38, 39], both 5hmC and 5mC-enriched regions were predominantly found in intragenic domains while 5hmC was enriched in gene bodies and at the 5 prime ends of genes. In hippocampus and ventricle, 5hmC was enriched in the gene bodies of actively transcribed genes. Interestingly, 5mC was enriched inside genes transcribed at low levels in hippocampus but not in heart. This is consistent with other studies that found global differences in methylation in brain versus blood [40]. Results from studies involving stem cells suggest that intragenic methylation correlates with active transcription [32]. Thus, the association of methylation with repressed genes in hippocampus suggests that this mark may have a repressive role in brain. Interestingly, intragenic methylation in brain has been linked to suppression of elongation and alternative splicing [41, 42].

We next examined the genome-wide distribution of 5mC and 5hmC relative to repetitive DNA. Both 5hmC and 5mC were detected in major repeat classes with greatest enrichment in SINEs and LINEs. These results are consistent with previous studies [43] and suggest that, like 5mC, 5hmC may play a role in repression of repeats and maintenance of genomic stability in these tissues. Interestingly, we observed a significant increase in 5hmC and decrease in 5mC at tRNA repeats in ventricle (relative to hippocampus) suggesting that the Tet/5hmC pathway may differentially regulate transcribed RNA repeats in the heart. Consistent with this idea, 5mC was significantly depleted at rRNA repeats in ventricle and there was a trend towards 5hmC depletion at rRNA repeats in hippocampus (*p* < 0.1). Similar regulation of tRNA-associated DMRs and DHRs was observed with subsets of the data indicating that epigenetic regulation of this class of repeats is global. Because regulation of tRNA transcription can regulate protein biogenesis this global epigenetic regulation may be biologically meaningful [44]. Thus, it is possible that differential Tet-mediated oxidation of tRNA and rRNA repeats in ventricle regulates transcription of these genes and potentially protein biogenesis.

Differential methylation in response to proton radiation was examined using merged regions at an FDR-adjusted *p* < 0.01. Because oxidation of 5mC into 5hmC is a sequential process believed to contribute to active demethylation, we examined the overlap of these two marks in hippocampus and heart. The inverse relationship between 5hmC and 5mC in heart we observed was anticipated because 5hmC is formed from 5 mC via sequential oxidation. Likewise, coordinated downregulation of 5hmC and 5mC in the heart is consistent with active demethylation occurring. The high-degree of overlap between regions that showed increased 5hmC and 5mC in brain is surprising because it suggests an active “maintenance” process. The brain is unique because it is the only tissue where high-levels of 5hmC are maintained throughout post-natal development [38, 45, 46]. Thus, we speculate that radiation-induced increases in 5hmC are similarly stabilized via an active process. Regardless, our data show that acute radiation exposure stably remodels the heart and brain epigenomes. Future studies with single nucleotide resolution could determine the degree to which *de novo* deposition of 5mC and 5hmC is regulated in a coordinated fashion.

The persistent epigenomic changes we observed were not random. Gene pathway analyses of domains that showed decreased 5mC in response to proton radiation revealed striking enrichment for key tissue-specific pathways, suggesting that epigenetic remodeling was associated with cellular differentiation responses. Moreover, these pathways were highly enriched for genes that are key regulators of cell-fate identity for brain and heart, respectively. For example in heart, SRF, Nkx2-5, Myocardin, and Myocardin-like are transcriptional master-regulators of heart development and differentiation that co-regulate overlapping gene pathways [47, 48]. All showed decreased accumulation of 5mC after exposure. Further, regions with decreased 5hmC in the ventricle were also enriched for muscle and heart-specific gene pathways. Given the role of these factors in cardiomyocyte and vascular differentiation, we hypothesize that epigenetic regulation of these genes represents a radiation-induced differentiation response. Interestingly, a recent study found that Tet2 was highly expressed in heart and that deletion of Tet2 resulted in hypermethylation of Myocardin and SRF and exacerbation of cardiovascular injury [49].

In hippocampus, genes associated with axon growth, neuronal differentiation, neurogenesis and synaptic proteins were enriched at domains with decreased 5mC in response to radiation. These results suggest epigenetic remodeling of pathways that regulate neuronal plasticity and may represent a compensatory response to damage. Hippocampal tissue also showed enrichment for genes linked to small G-protein signaling and cytoskeletal remodeling at regions associated with increased 5hmC. The association with cytoskeletal remodeling is consistent with alterations in spine measures seen 30 days following proton irradiation [50] and 60 days following ^56^Fe irradiation [51].

Most importantly, our data highlight that proton irradiation generates a tissue-specific response that targets key regulators of differentiation and plasticity in heart and brain. As noted in the Introduction, ionizing radiation is just one of many forms of environmental exposures. A logical, albeit speculative, extension of our data is that other forms of environmental exposures also cause tissue-specific epigenomic responses, at least in post-mitotic cells. Presumably, in addition to tissue specific responses, exposure specific responses would also be observed because different agents and stressors induce different cellular responses. If so, our cells are repositories of exposures accumulated over a lifetime.

## Conclusion

In summary, our data present clear evidence of tissue-dependent epigenetic effects of proton irradiation, as well as some shared effects that are consistent with a common response to radiation damage. The gene methylation data in both tissues revealed significant major pathway changes that are related to known pathophysiologic processes. The tissue-dependent results are unique in the context of response to radiation and, combined with the major pathway changes identified, support the power of this approach.

## Methods

### Animals and study design

Six-month-old C57BL/6J male mice (*n* = 10 mice) were obtained from Jackson Laboratories, Bar Harbor Maine. The mice were shipped from Jackson Laboratories to Brookhaven National Laboratory (BNL), Upton, Long Island, New York, and allowed to accommodate to the housing facility there for one week. Subsequently, the mice were irradiated with 1 Gy of 150 MeV protons or sham-irradiated. For irradiation, mice were loaded into 8 x 3 x 3 cm plastic square enclosures with air holes and placed in a foam fixture in the beam line of the NASA Space Radiation Laboratory (NSRL). They were exposed to a rectangular beam of approximately 20 x 20 cm. The focused beam of high-energy was generated by the Booster accelerator at BNL and transferred to the experimental beam line at the NSRL facility. Dose calibration was performed to ensure that the desired dose was delivered. Sham-irradiated mice were placed into the plastic enclosures for the same time as the irradiated mice. Mice were randomly assigned to the experimental groups. One week after the irradiation or sham-irradiation, the mice were shipped to Oregon Health & Science University (OHSU) and were killed by cervical dislocation for analyses 22 weeks after the irradiation date. The hippocampus of one hemibrain and left ventricle of 10 mice were divided in separate tissues for DNA methylation analyses. All protocols were reviewed and approved by the Institutional Animal Care and Use Committees (IACUC) of OHSU and BNL and were in compliance with all Federal regulations.

### DNA methylation sequencing

DNA was isolated from the left ventricle and hippocampus. Antibodies against 5mC and 5hmC were used to immunoprecipitate DNA preparations for methyl-DNA immunoprecipitation (meDIP) and hydroxymethyl-DNA immuno precipitation, respectively, from eight pools of tissues (2 x 2 pools of hippocampal tissues and 2 x 2 pools of left ventricle tissues, or 2 pools/tissue/radiation condition) (see Fig. 1 for a diagram of the protocol). The antibodies used against 5mC and 5hmC do not cross react. These antibodies were used to precipitate genomic regions that are enriched for either 5mC or 5hmC. Following immunoprecipitation, high throughput genomic sequencing was used to identify these enriched genomic regions. For DIP-Seq library preparation, RNAse-treated DNA was isolated using the Qiagen Allprep DNA/RNA protocol. The DNA was sonicated using a Cole Parmer CPX-132 sonicator (75 % amplitude, 3x10’) and polished using the DNA terminator end repair kit (Lucigen). DNA fragments were A-tailed using Klenow exo-(Epicenter) and ligated to un-methylated HT TrueSeq indexed adapters and purified. The resulting purified DNA was denatured at 95 C, resuspended in 100 ul of DIP IP buffer, and immunoprecipitated with 1 μg of the highly specific 5-methylcytosine antibody (Eurogentec) or 2 ul of 5-hydroxymethylcytosine (Active Motif) antibody and Dynal anti-mouse IgG beads. Beads were rinsed 7 times with IP buffer, eluted with 1 % SDS at room temperature and the eluted DNA is purified and subjected to limited amplification (~18 cycles). Libraries were sequenced on the HiSeq2000 platform at the OHSU Massively Parallel Sequencing Shared Resource or the Oregon State University Center for Genome Research and Biocomputing.

### Bioinformatics and statistics

35 bp single read sequence data was mapped to the mouse reference genome (UCSC mm9) using the Bowtie algorithm using standard flags and allowing 2 mismatches [52]. Sequences that map to a single location were selected and domains enriched for 5mC or 5hmC were selected using a parameter-optimized Monte-Carlo-based segmentation algorithm [53]. A 1000 bp sliding-window was used based on iterative analyses that maximized the number of enriched regions. A comparison of different high-throughput sequencing based methods to study DNA methylation concluded that MeDIP-Seq covers ~ 67 % of genomic CpGs [54].

### Statistical analyses

For statistical comparisons of biological samples, regions of methylation enrichment were merged and differences in methylation interrogated with FDR-adjusted chi-square or negative binomial statistics [55]. Statistical and visualization studies involved the R programming language and Bioconductor packages [56]. Gene ontology analyses utilized the bioconductor Goseq package, which adjusts for RNA-Seq length bias artifacts [57]. For gene ontology analyses the top 2000 DMRs or DHRs (FDR-adjusted p < 0.01) within 50kb of a transcriptional start site were non-redundantly annotated. Pathway analyses involved standard bioconductor packages (e.g. cmap, keggraph, gsea). DIP sequence-tag heatmaps were generated in R by plotting median-normalized DIP-Seq tag density in gene bodies and indicated flanking regions with color-maps scaled to the 80 % quantile. Statistical analyses of pathway data were conducted via FDR-adjusted Fisher exact or KS-tests. We considered *p* < 0.05 as statistically significant.

## Additional files

Additional file 1: Figure S1. DNA was isolated and changes in the levels and distributions of 5mC and 5hmC from proton exposure were determined using me-DIP (5mC) and hme-DIP (5-hmC), respectively. The 5mC and 5hmC antibodies were highly specific, with no detectable cross-reactivity by DNA dot blot. DIP-Seq libraries were generated (2 per tissue/radiation condition) each with greater than 30 million reads. (TIFF 919 kb) Additional file 2: Figure S2. Heatmaps show sequence density for hippocampal 5hmC DIP-Seq experiments generated in this manuscript and in Szulwach et. al [31] in RefSeq genes ranked by hippocampal RNA-Seq gene expression. The data was scaled to the 80th quantile and upstream and downstream 15 kb regions are depicted (not to scale). The inset dot blot illustrates the specificity of the antibodies used using fully methylated or hydroxymethylated DNA. (TIFF 1560 kb) Additional file 3: Table S1, S2 and S3. Significant DMRs and DHMRs annotated with the closest RefSeq transcriptional start site. (XLS 9469 kb) Additional file 4: Figure S3. Bar graph depicts gene ontology categories significantly enriched in the indicated tissue-specific differentially methylated regions (FDR-adjusted *p* < 0.01). (TIFF 2406 kb) Additional file 5: Figure S4. Kegg pathway diagrams illustrate overrepresented gene-associated DMRs decreased by radiation in ventricle. (FDR-adusted *p* < 0.001). (TIFF 1298 kb) Additional file 6: Figure S5. Kegg pathway diagram illustrate overrepresented gene-associated DMRs decreased by radiation in hippocampus. (FDR-adusted *p* < 0.001). (JPG 121 kb)
