# Supplementary figures and images for: Proton irradiation induces persistent and tissue-specific DNA methylation changes in the left ventricle and hippocampus
Source: BMC Genomics. 2016 Mar 31;17:273. doi: 10.1186/s12864-016-2581-x (PMC4815246; doi:10.1186/s12864-016-2581-x)

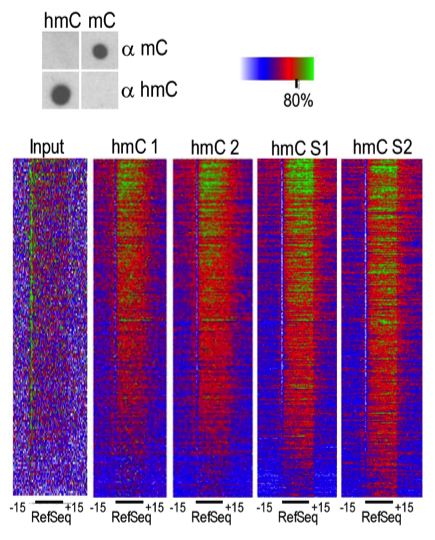

Supplement: Additional file 1: Figure S1. — DNA was isolated and changes in the levels and distributions of 5mC and 5hmC from proton exposure were determined using me-DIP (5mC) and hme-DIP (5-hmC), respectively. The 5mC and 5hmC antibodies were highly specific, with no detectable cross-reactivity by DNA dot blot. DIP-Seq libraries were generated (2 per tissue/radiation condition) each with greater than 30 million reads. (TIFF 919 kb) [file 12864_2016_2581_MOESM1_ESM.tiff]

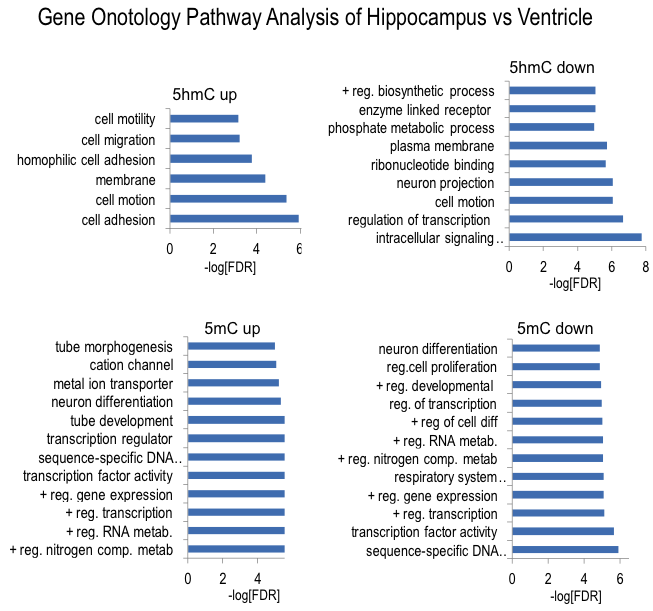

Supplement: Additional file 2: Figure S2. — Heatmaps show sequence density for hippocampal 5hmC DIP-Seq experiments generated in this manuscript and in Szulwach et. al [31] in RefSeq genes ranked by hippocampal RNA-Seq gene expression. The data was scaled to the 80th quantile and upstream and downstream 15 kb regions are depicted (not to scale). The inset dot blot illustrates the specificity of the antibodies used using fully methylated or hydroxymethylated DNA. (TIFF 1560 kb) [file 12864_2016_2581_MOESM2_ESM.tiff]

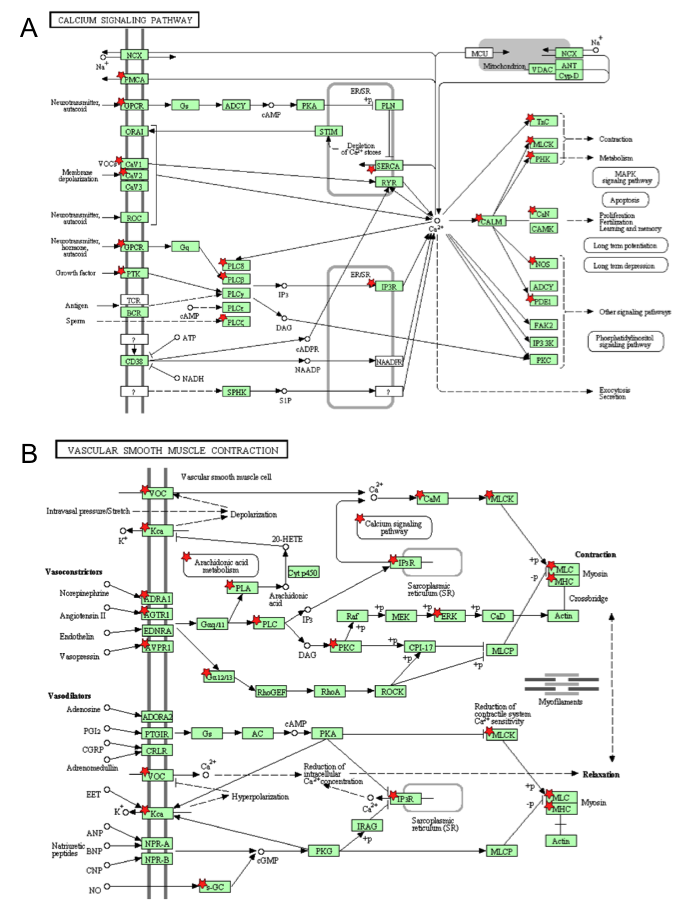

Supplement: Additional file 4: Figure S3. — Bar graph depicts gene ontology categories significantly enriched in the indicated tissue-specific differentially methylated regions (FDR-adjusted p < 0.01). (TIFF 2406 kb) [file 12864_2016_2581_MOESM4_ESM.tiff]

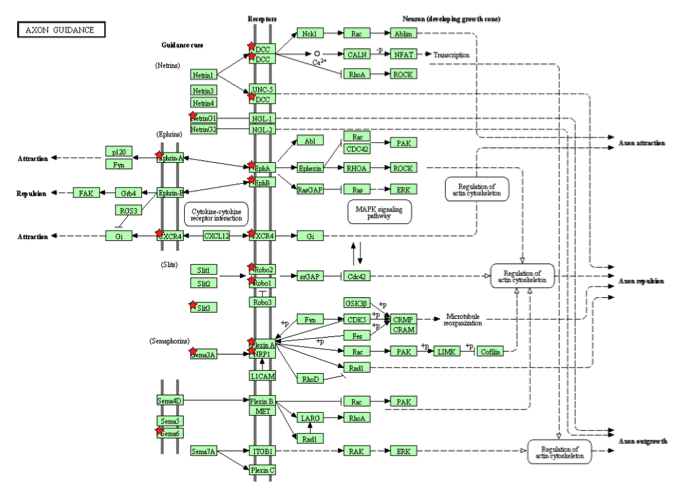

Supplement: Additional file 5: Figure S4. — Kegg pathway diagrams illustrate overrepresented gene-associated DMRs decreased by radiation in ventricle. (FDR-adusted p < 0.001). (TIFF 1298 kb) [file 12864_2016_2581_MOESM5_ESM.tiff]

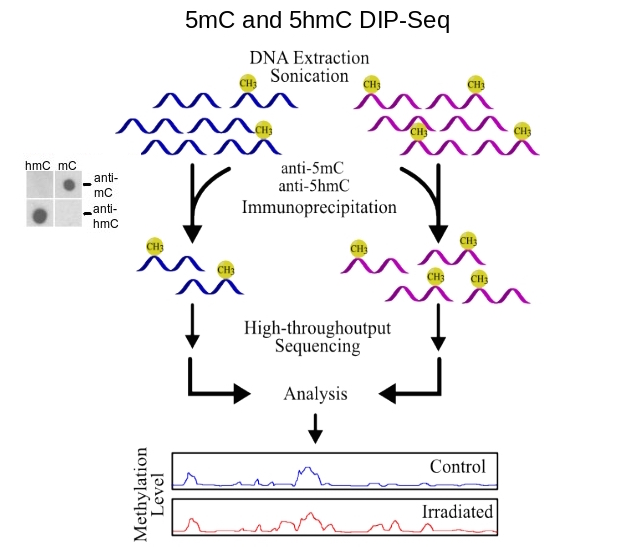

Supplement: Additional file 6: Figure S5. — Kegg pathway diagram illustrate overrepresented gene-associated DMRs decreased by radiation in hippocampus. (FDR-adusted p < 0.001). (JPG 121 kb) [file 12864_2016_2581_MOESM6_ESM.jpg]
